# *Hirsutella sinensis* mycelium attenuates bleomycin-induced pulmonary inflammation and fibrosis *in vivo*

**DOI:** 10.1038/srep15282

**Published:** 2015-10-26

**Authors:** Tsung-Teng Huang, Hsin-Chih Lai, Yun-Fei Ko, David M. Ojcius, Ying-Wei Lan, Jan Martel, John D. Young, Kowit-Yu Chong

**Affiliations:** 1 Center for Molecular and Clinical Immunology, College of Medicine, Chang Gung University, Taoyuan 33302, Taiwan, ROC; 2 Department of Medical Biotechnology and Laboratory Science, College of Medicine, Chang Gung University, Taoyuan 33302, Taiwan, ROC; 3 Laboratory of Nanomaterials, College of Medicine, Chang Gung University, Taoyuan 33302, Taiwan, ROC; 4 Biochemical Engineering Research Center, Ming Chi University of Technology, New Taipei City 24301, Taiwan, ROC; 5 Chang Gung Biotechnology Corporation, Taipei 10508, Taiwan, ROC; 6 Department of Biomedical Sciences, University of the Pacific, Arthur Dugoni School of Dentistry, San Francisco, CA 94103, USA; 7 Graduate Institute of Biomedical Sciences, Division of Biotechnology, College of Medicine, Chang Gung University, Taoyuan 33302, Taiwan, ROC; 8 Laboratory of Cellular Physiology and Immunology, Rockefeller University, New York, NY 10021, USA; 9 Department of Family Medicine, Chang Gung Memorial Hospital-Linkou, Taoyuan 33305, Taiwan, ROC

## Abstract

*Hirsutella sinensis* mycelium (HSM), the anamorph of *Cordyceps sinensis*, is a traditional Chinese medicine that has been shown to possess various pharmacological properties. We previously reported that this fungus suppresses interleukin-1β and IL-18 secretion by inhibiting both canonical and non-canonical inflammasomes in human macrophages. However, whether HSM may be used to prevent lung fibrosis and the mechanism underlying this activity remain unclear. Our results show that pretreatment with HSM inhibits TGF-β1–induced expression of fibronectin and α-SMA in lung fibroblasts. HSM also restores superoxide dismutase expression in TGF-β1–treated lung fibroblasts and inhibits reactive oxygen species production in lung epithelial cells. Furthermore, HSM pretreatment markedly reduces bleomycin–induced lung injury and fibrosis in mice. Accordingly, HSM reduces inflammatory cell accumulation in bronchoalveolar lavage fluid and proinflammatory cytokines levels in lung tissues. The HSM extract also significantly reduces TGF-β1 in lung tissues, and this effect is accompanied by decreased collagen 3α1 and α-SMA levels. Moreover, HSM reduces expression of the NLRP3 inflammasome and P2X_7_R in lung tissues, whereas it enhances expression of superoxide dismutase. These findings suggest that HSM may be used for the treatment of pulmonary inflammation and fibrosis.

Idiopathic pulmonary fibrosis (IPF) is a progressive and irreversible lung disease of unclear etiology that predominantly occurs in middle-aged and elderly adults^1^,^2^. Although several drugs have been developed to treat this condition, the five-year survival rate of IPF remains less than 50% and the drugs’ serious adverse effects pose problems during long-term treatment. For these reasons, herbal remedies and traditional Chinese medicines have emerged as attractive alternatives for the treatment of fibrotic lung disease.

IPF is characterized by failure of alveolar re-epithelialization, differentiation of fibroblasts into myofibroblasts, excessive deposition of extracellular matrix (ECM), and distortion of normal lung architecture, processes which ultimately result in respiratory failure^3^. Bleomycin (BLM) is a chemotherapeutic agent known to produce interstitial pulmonary fibrosis in humans and experimental animal models^4^,^5^. BLM is widely used to induce lung fibrosis in animal models and to identify the pathogenic mechanisms underlying this condition as well as novel therapeutic strategies. Previous studies have shown that many proinflammatory cytokines and profibrotic growth factors, including interleukin-1 beta (IL-1β), IL-6, IL-18, tumor necrosis factor-alpha (TNF-α), and transforming growth factor-beta 1 (TGF-β1) are involved in pulmonary inflammation and fibrosis^6^,^7^,^8^.

TGF-β1 is one of the most studied fibrogenic cytokines that play a role in induction and development of pulmonary fibrosis^9^. This cytokine initiates the differentiation of fibroblasts into active myofibroblasts, which promote excessive collagen and ECM deposition, and contribute to the recruitment of inflammatory cells^10^. Differentiation of fibroblasts into myofibroblasts is identified by the expression of α-smooth muscle actin (α-SMA), and both fibroblasts and myofibroblasts are primary sources of ECM proteins such as fibronectin and collagen^11^. In addition, TGF-β1 induces fibroblast-to-myofibroblast differentiation by activating the Smad2/3 and Akt signaling pathways^12^,^13^. Enhanced expression of TGF-β1 in the lungs has been detected in lung fibrosis animal models and IPF patients^14^,^15^. These observations suggest that inhibition of the fibrogenic cytokine TGF-β1 represents a potential strategy for pulmonary fibrosis therapy.

IL-1β and IL-18 are produced intracellularly from the inactive precursors, pro-IL-1β and pro-IL-18^16^. Mature IL-1β and IL-18 are secreted following cleavage of pro-IL-1β and pro-IL-18 by the cysteine-protease, caspase-1, originally identified as IL-1β converting enzyme. Activation of caspase-1 requires the assembly and activation of a cytosolic protein complex called the inflammasome, which consists of a nucleotide-binding oligomerization domain (NOD)-like receptor (NLR) family member, the adaptor protein apoptosis-associated speck-like protein containing a caspase-recruitment domain (ASC), and pro-caspase-1^17^. Once assembled, the inflammasome activates caspase-1, which then induces the cleavage and maturation of pro-IL-1β and pro-IL-18. The NLRP3 inflammasome (for NOD-like receptor, pyrin domain containing-3) is activated by a wide range of stimuli, including ATP, monosodium urate crystals, cholesterol crystals, UV irradiation, microbes, silica, asbestos, and amyloid-β^18^,^19^,^20^,^21^,^22^. A recent study performed using gene knockout mice suggests that the NLRP3 inflammasome mediates the development of fibrosis in systemic sclerosis^23^. Furthermore, NLRP3 appears to play a key role in promoting TGF-β1 signaling and Smad2/3 activation in kidney epithelial cells^24^.

BLM treatment has been shown to increase the production of reactive oxygen species (ROS) and induce the development of lung fibrosis^25^. In addition, ROS have been shown in many cases to trigger NLRP3 inflammasome activation^18^. To counteract oxidative stress induced by ROS, lung tissues express several antioxidant enzymes, such as superoxide dismutases (SODs), which convert superoxide radicals to hydrogen peroxide^26^. SOD expression is markedly reduced in BLM-induced lung fibrosis^27^. Another study showed that increased ATP levels were found in bronchoalveolar lavage fluid (BALF) of patients with IPF and in BLM–treated mice. Furthermore, the purinergic receptor P2X_7_R is activated by ATP, which is released by injured lung cells following BLM treatment, leading to activation of the NLRP3 inflammasome, cleavage and secretion of IL-1β, and development of lung fibrosis^28^.

*Cordyceps sinensis* (also known as *Ophiocordyceps sinensis*) is a parasitic fungus that infects larvae of ghost moths^29^. Used for centuries in Asia as one of the most valued traditional Chinese medicines, *C. sinensis* has been shown in recent years to possess various therapeutic functions, including anti-cancer, anti-diabetic, anti-inflammatory, immunomodulatory, and anti-oxidant effects^30^,^31^,^32^,^33^. Owing to the rarity of wild *C. sinensis* fruiting bodies, which grow exclusively on the Tibetan-Qinghai plateau and the Himalayas, the cultivation of *Hirsutella sinensis*, the anamorphic mycelial stage of natural *C. sinensis*, has emerged as an attractive substitute for the preparation of health supplements^34^. In our previous studies, we showed that an ethanol extract of *H. sinensis* mycelium (HSM) suppresses IL-1β and IL-18 secretion by inhibiting both canonical and non-canonical inflammasomes in human macrophages^35^. Recently, *C. sinensis* was shown to reduce liver fibrosis by inhibiting TGF-β1, α-SMA, collagen type I and III expression, as well as TGF-β1–mediated Smad2/3 signaling^36^,^37^. Other studies have shown that *C. sinensis* markedly attenuates the decline of renal function and renal fibrosis in subtotal nephrectomy rats—effects that were associated with reduced expression of the fibrogenic marker α-SMA and inhibition of TGF-β1/Smad signaling^38^. In addition, Chen *et al.* observed that *C. sinensis* fruiting bodies reduce lung fibrosis symptoms in rats^39^. However, the effects of HSM ethanol extract on lung fibrosis and the possible mechanism of action of this fungal remedy have not been investigated.

The objectives of the present study were to investigate whether oral intake of HSM ethanol extract attenuates lung inflammation and fibrosis in mice, in addition to examining the molecular mechanisms of this protective effect. Our results show that HSM protects mice against BLM–induced lung inflammation and fibrosis by inhibiting NLRP3 inflammasome activation, ROS production, and TGF-β1–induced Smad2/3 and Akt signaling.

## Methods

### Chemicals and reagents

BLM sulfate purified from *Streptomyces verticillus* was obtained from Sigma-Aldrich (St. Louis, MO, USA). Recombinant human TGF-β1 was purchased from R&D Systems (Minneapolis, MN, USA). Minimum essential medium (MEM), Dulbecco’s modified Eagle’s medium (DMEM)/Ham’s F12, fetal bovine serum (FBS), penicillin, and streptomycin were purchased from Life Technologies (Grand Island, NY, USA). Antibodies against Akt, phospho-Akt, Smad2/3, and phospho-Smad2/3 were obtained from Cell Signaling Technology (Danvers, MA, USA); antibodies against ASC, caspase-1, fibronectin, IL-1β, IL-18, NLRP3, SOD, and β-actin were purchased from Santa Cruz Biotechnology (Dallas, TX, USA). Antibodies raised against TGF-β1 and P2X _7_R were purchased from Abcam (Cambridge, MA, USA). Antibodies against α-SMA and collagen, type III, alpha 1 chain (collagen 3α1) were obtained, respectively, from Sigma-Aldrich and Acris Antibodies (San Diego, CA, USA). Horseradish peroxidase-conjugated anti-rabbit and anti-mouse secondary antibodies were obtained from Santa Cruz Biotechnology.

### Preparation of HSM ethanol extract

The *H. sinensis* strain originally isolated and characterized at Chang Gung Biotechnology (Taipei, Taiwan) was identified based on DNA analysis of internal transcribed spacers^40^. The ethanol extract was prepared as described earlier^35^.

### Cell culture and treatment

Fetal human MRC-5 lung fibroblasts (BCRC-60023, Bioresource Collection and Research Center, Hsinchu, Taiwan) and mouse MLE12 lung epithelial cells (CRL-2110, American Type Culture Collection, Manassas, VA, USA) were maintained in MEM and DMEM/Ham’s F12 supplemented with 10% FBS, 100 units/ml of penicillin, and 100 μg/ml of streptomycin (Life Technologies). MRC-5 cells were cultured in 6-well plates at a density of 2 × 10^5^ cells/well at 37 °C in a humidified cell culture incubator with 5% CO_2_. Before experiments, MRC-5 cells were washed with phosphate buffered saline (PBS) and serum-starved for 24 h, prior to stimulation with HSM extract (1% or 2%, v/v) or with 2% ethanol as a control for the time indicated. TGF-β1 (5 ng/ml) was used as positive control to induce myofibroblast differentiation. At the end of the exposure period, the cells were lysed to isolate proteins for Western blot analysis.

### Cell viability assay

Cell viability was determined using a commercial MTT-based *in vitro* toxicology assay (Sigma-Aldrich), which detects viable cells colorimetrically based on the production of purple formazan. MRC-5 and MLE12 cells were initially seeded in 96-well plates at a density of 2 × 10^4^ cells/well and 3 × 10^4^ cells/well, respectively, prior to incubation for 24 h at 37 °C. Cell culture media were replaced by complete media containing the indicated concentration of HSM ethanol extract, prior to incubation for 24 h or 48 h. After incubation, 10 μl of MTT (5 mg/ml) were added to each well, and the plates were incubated for 4 h at 37 °C. The content of each well was eluted and the precipitate was dissolved in 100 μl of the MTT solubilization solution. Absorbance was read at 570 nm using a SpectraMax M5 multi-mode microplate reader (Molecular Devices, Sunnyvale, CA, USA). Cell viability (%) was calculated as the ratio of surviving cells in the HSM-treated group divided by that of the control group. All treated samples and controls were tested in triplicate.

### Murine model of bleomycin-induced lung fibrosis

Eight-week-old male C57BL/6 mice (average weight of 22–25 g) were purchased from the National Laboratory Animal Center (Taipei, Taiwan). The mice were maintained in an air-conditioned animal facility under constant temperature and humidity with a 12-h day-night cycle and food and water ad libitum. All experimental procedures were conducted under the Institutional Animal Care and Use Committee protocols (ID number: CGU12–024) approved by Chang Gung University and in compliance with the Animal Welfare Act and the principles set forth in the Guide for the Care and Use of Laboratory Animals National Research Council, National Academies Press, 1996. Mice were randomly divided into four treatment groups: (1) PBS+EtOH: intratracheal PBS plus oral ethanol; (2) PBS+HSM: intratracheal PBS plus oral HSM ethanol extract; (3) BLM+EtOH: intratracheal BLM plus oral ethanol; (4) BLM+HSM: intratracheal BLM plus oral HSM ethanol extract. The BLM sulfate stock was prepared by dissolving the compound in sterile PBS at 10 mg/ml and storing small aliquots at 4 °C. Mice were anesthetized using isoflurane (Abbott Laboratories, Abbott Park, IL, USA) and BLM was dissolved in 50 μl of PBS and administered intratracheally (1.5 mg/kg) on day 0 as previously described^41^ while control animals received an equal volume of sterile PBS. The 10% HSM extract used in this experiment was prepared by dissolving HSM ethanol extract in 0.1 ml of sterile PBS. HSM extract (10%) or control ethanol (10%) was administered by oral gavage once daily for 7 days prior to the intratracheal instillation of BLM or PBS and until conclusion of the experiments. On day 7 or 21, animals were sacrificed using 2.5% avertin (Sigma-Aldrich).

### Bronchoalveolar lavage fluid (BALF)

Seven days after BLM treatment, five mice in each group were killed and BALF was obtained by washing three times with 1 ml of cold sterile PBS through a tracheal cannula. BALF samples were centrifuged at 1,000 × *g* for 10 min at 4 °C, and the cell pellet was resuspended in PBS. Total BALF cell count was performed using a hemocytometer. The cells were cytospun onto a microscope slide and stained with Wright-Giemsa (Sigma-Aldrich) for cell classification. Percentages of BALF macrophages, neutrophils and lymphocytes were obtained by counting leukocytes under light microscopy.

### Histopathological analysis

Tissue samples from the left lung of sacrificed mice were collected and immediately fixed with 4% paraformaldehyde before embedding with paraffin wax and routine processing. Serial paraffin sections (4 μm) were prepared using a rotator microtome, and deparaffinized tissue sections were stained with hematoxylin and eosin (H&E, Sigma-Aldrich) to evaluate morphological changes in lungs.

The severity of lung fibrosis was evaluated using Masson’s trichrome staining (Trichrome Stain Kit, Sigma-Aldrich). Masson’s trichrome staining was used to differentiate collagen from other fibers, staining nuclei in black, cytoplasm and muscles in red, and collagen in blue. To determine the severity of lung fibrosis, each successive field was semi-quantitatively assessed based on previous protocols^41^. Criteria for grading lung fibrosis were as follows: Grade 0, normal lung; Grade 1, minimal fibrous thickening of alveolar or bronchiolar walls; Grade 2–3, moderate thickening of walls without obvious damage to lung architecture; Grade 4–5, increased fibrosis with definite damage to lung structure and formation of fibrous bands or small fibrous masses; Grade 6–7, severe distortion of lung structures and large fibrous areas (honeycomb lungs’ were classified in this category); Grade 8, total fibrous obliteration of fields. The mean score of all fields was used as the fibrosis score of that lung section.

### Immunohistochemical staining of α-SMA, ASC, and NLRP3

Paraffin-fixed sections were treated with xylene and a graded ethanol series to remove paraffin and for rehydration. The lung sections were microwave-heated (750 W, three 5-minute cycles) in citrate buffer (10 mM of sodium citrate, pH 6.0) for antigen retrieval. Endogenous peroxidase was quenched using 3% hydrogen peroxide (H_2_O_2_) for 15 min at room temperature. The sections were immunostained with primary antibodies against α-SMA, ASC, or NLRP3 overnight at 4 °C, prior to incubation in EnVision Detection Systems (Dako, Glostrup, Denmark) according to the manufacturer’s instructions. The bound antibodies were visualized using diaminobenzidine, and all slides were counterstained with hematoxylin, dehydrated by gradually increasing the concentration of ethanol, cleared in xylene, and mounted in glycerol-gelatin. Images from stained slides were acquired with HistoFAXS (Tissue FAX Plus; Tissue Gnostics, Vienna, Austria).

### RNA isolation and quantitative real-time RT-PCR

Total RNA from lung tissues were isolated using RNeasy Mini Kit (Qiagen, Valencia, CA, USA) according to the protocol provided by the manufacturer. One microgram of total RNA was reverse-transcribed in a solution (final volume of 20 μl) containing oligo dT primer, dNTP, and reverse transcriptase (SuperScript III, Invitrogen, Carlsbad, CA, USA). Sequences of the mouse gene-specific primers used in this study are listed in Table 1. Quantitative real-time PCR was performed with SYBR Green Master Mix (Roche, Mannheim, Germany) as described previously^42^. Relative quantification of gene expression was assessed using a mathematical model provided by the manufacturer. Relative mRNA levels were normalized to β-actin mRNA. Each fold expression is based on an average of at least 3–5 biological replicates per treatment group.

### Western blot analysis

Cellular proteins were isolated by resuspending cells or lung tissues in RIPA lysis buffer (50 mM Tris-HCl, pH 7.4, 150 mM NaCl, 0.25% deoxycholic acid, 1% Nonidet P-40, 1 mM EDTA) (Millipore, Billerica, MA, USA) containing a protease inhibitor cocktail (Roche). Total protein concentrations were assessed using a commercial Bradford assay (Bio-Rad, Hercules, CA, USA). Equal amounts of protein were loaded and separated onto 8-to-12% SDS-PAGE. After electrophoresis, the resolved proteins were transferred to PVDF membranes (Millipore). Membranes were blocked with 5% defatted milk in TBS-T (0.1% Tween-20 in 1 × TBS, pH 7.4) for 1 h at room temperature, followed by incubation overnight with primary antibodies at 4 °C. After washing steps, the membranes were incubated with horseradish peroxidase-conjugated secondary antibodies for 1 h at room temperature, and protein signals were revealed using enhanced chemiluminescence reagents (Millipore). To adjust for minor loading differences, the optical density of each protein was normalized against β-actin. For Western blot analysis of lung tissues, 3 to 5 mice were analyzed for each group.

### Measurement of ROS production

ROS production in MRC-5 cells and MLE12 cells was measured using the Total ROS/Superoxide detection kit (Enzo Life Sciences, Farmingdale, NY, USA). Briefly, MRC-5 cells and MLE12 cells were seeded in 96-well black wall/clear bottom plates at a density of 2 × 10^4^ cells/well and 3 × 10^4^ cells/well, respectively, and allowed to adhere for 24 h. Cell culture media were replaced by complete media containing the indicated concentration of HSM ethanol extract for 24 h, prior to treatment with BLM (2.5 μM) for 24 h. In some experiments, the cells were treated with pyocyanin (200 μM) for 30 min at 37 °C. After treatment, the cells were washed with 200 μl of 1× washing buffer and loaded with 100 μl of ROS/Superoxide detection reagent, prior to incubation at 37 °C for 1 h. Absorbance was read at a wavelength of 520 nm after excitation at 488 nm. Intracellular ROS production was determined based on the increase of relative fluorescence intensity.

### Statistical analysis

All results are shown as mean values ± standard error of the mean (SEM) from at least three independent experiments with duplicate of each condition. Comparisons for multiple groups were performed by one-way analysis of variance (ANOVA) followed by Dunnett’s post hoc test. Differences between two means were evaluated using the two-tailed Student’s *t*-test. For all analyses, a *p* value <0.05 was considered statistically significant.

## Results

### HSM ethanol extract inhibits TGF-β1–induced differentiation of lung fibroblasts into myofibroblasts

**Figure 1:**
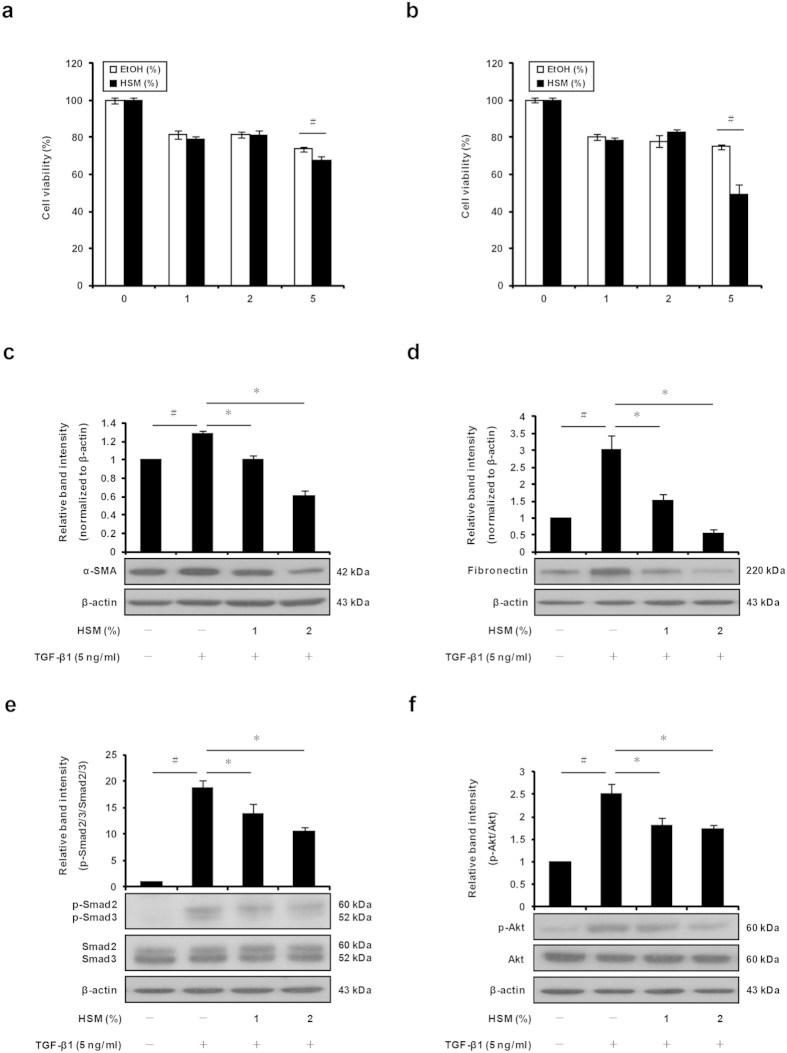
Anti-fibrotic effects of HSM ethanol extract on the production of myofibroblast marker proteins α-SMA and fibronectin through suppression of Smad2/3 and Akt signaling pathways in MRC-5 human lung fibroblasts. Cells were treated with various concentrations (1, 2, and 5%) of HSM extract for 24 h (**a**) or 48 h (**b**), and cell viability was measured using the MTT assay. (**c,d**) Cells were serum-starved overnight, before treatment with varying concentrations (1 and 2%) of HSM extract for 24 h and incubation with TGF-β1 (5 ng/ml) for 24 h. Expression of the myofibroblast markers α-SMA and fibronectin were evaluated in whole cell lysates by Western blotting. β-actin was used as a loading control. Relative protein levels were quantified by scanning densitometry and were normalized to β-actin. (**e,f**) Cells were pretreated with HSM extract (1 and 2%), followed by treatment with TGF-β1 (5 ng/ml) for another 6 h. Phosphorylated and total Smad2/3 and Akt were measured by Western blotting against phospho-Smad2/3, Smad2/3, phospho-Akt and Akt. Blots were analyzed by densitometry and the results were expressed as relative units. Data shown represent means ± SEM of three experiments performed in duplicate. ^#^*P* < 0.05 versus untreated or ethanol–treated cells. *P < 0.05 versus control (TGF-β1–treated) cells.

We first examined the effect of HSM on the viability of lung fibroblasts. Human MRC-5 lung fibroblasts were treated with HSM (1, 2, or 5%) or the vehicle (ethanol) for 24 h or 48 h, and cell viability was assessed using the MTT assay. As shown in Fig. 1a,b, HSM at 5% significantly reduced cell viability after 24 h and 48 h of incubation compared to control ethanol. Based on this observation, the experiments in cultured lung fibroblasts were conducted using 1 and 2% of HSM extract.

Myofibroblast accumulation represents an important sign of lung and liver fibrosis^43^. Fibroblasts treated with the profibrogenic cytokine TGF-β1 show increased expression of α-SMA and fibronectin, which are important markers of myofibroblast differentiation. We used an *in vitro* model of fibroblast activation to examine the effects of HSM on TGF-β1–induced fibroblast differentiation. Serum–starved MRC-5 fibroblasts were treated with HSM extract (0–2%) for 24 h, followed by exposure to TGF-β1 (5 ng/ml) for 24 h. Cell differentiation was assessed by measuring α-SMA and fibronectin using Western blotting. While α-SMA and fibronectin protein expression was significantly induced by TGF-β1, pretreatment with HSM reduced α-SMA and fibronectin levels in a dose-dependent manner (Fig. 1c,d).

### HSM inhibits TGF-β1–induced phosphorylation of Smad2/3 and Akt in lung fibroblasts

TGF-β1 induces fibroblast-to-myofibroblast differentiation by activating Smad–dependent and Smad–independent responses, including PI3K/Akt–mediated signaling^12^,^13^. To determine the signaling pathways affected by HSM, we pretreated lung fibroblasts with the HSM extract for 24 h, prior to TGF-β1 treatment (5 ng/ml) for 6 h. TGF-β1 induced a significant increase of phosphorylated Smad2/3 and Akt in lung fibroblasts, whereas total Smad2/3 and Akt protein levels remained constant (Fig. 1e,f). Pretreatment with HSM for 24 h significantly decreased phosphorylated Smad2/3 and Akt protein levels in TGF-β1–treated cells, while total Smad2/3 and Akt were not affected (Fig. 1e,f). These results indicate that the HSM ethanol extract inhibits the effects of TGF-β1 on Smad2/3 and Akt signaling pathways.

### HSM attenuates profibrotic and inflammatory responses in a murine model of BLM–induced pulmonary fibrosis

**Figure 2:**
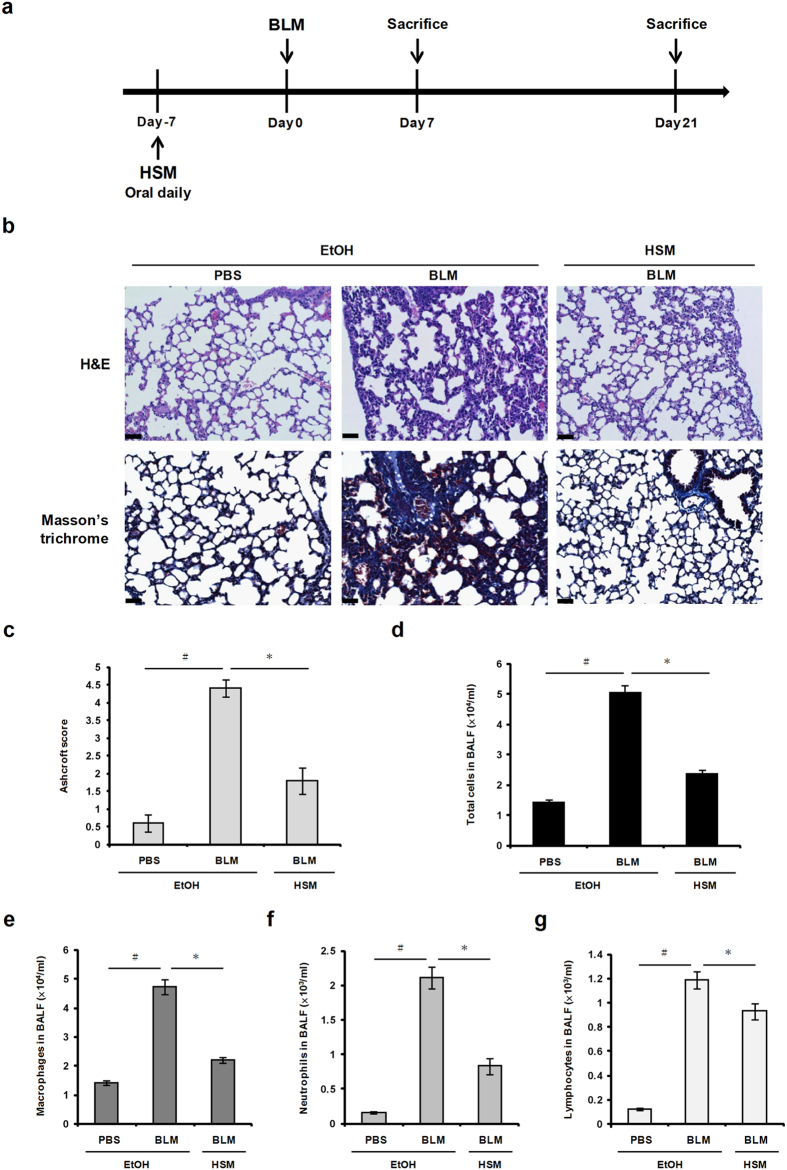
HSM alleviates BLM–induced lung fibrosis and inflammatory cells accumulation in mice. (**a**) Experimental design: Mice were instilled intratracheally with BLM (1.5 mg/kg in 50 μl of PBS). The treatment group received HSM ethanol extract (10%) orally for a week before BLM instillation. The mice were sacrificed on day 7 and 21 following BLM instillation, and lung samples were collected for further analysis. (**b**) Paraffin sections from lung tissues of the mice on day 21 were stained with H&E or Masson’s trichrome. Scale bars = 50 μm. (**c**) Quantitative examination of the effects of HSM ethanol extract on BLM–induced lung fibrosis. (**d–g**) BALF was collected 7 days after BLM treatment to quantify total cells, macrophages, neutrophils, and lymphocytes. The values shown represent means ± SEM (n = 5 in each group). ^#^*P* < 0.05 versus PBS + EtOH group. *P < 0.05 versus BLM + EtOH group.

BLM–induced lung fibrosis is a well-established animal model in which oral intake of BLM results in airway epithelial cells damage, inflammation, fibroblast proliferation and differentiation, and extracellular collagen deposition in lung tissues^5^. Figure 2a illustrates the strategy used to investigate the effects of HSM in male C57BL/6 mice treated with BLM. In the control group (PBS + EtOH), no apparent histological change was detected in lung tissues (Fig. 2b). In contrast, the lung parenchyma of BLM–treated mice showed increasing alveolar wall thickness, inflammatory cell infiltration, vascular congestion, and alveolar space collapse (Fig. 2b). These histopathological changes were improved by HSM pretreatment (Fig. 2b).

To examine whether HSM produces anti-fibrotic effects, we used Masson’s trichrome staining to assess collagen deposition in lung tissues, 21 days following BLM administration. Lung sections from the control group showed a small amount of collagen fibers (stained blue) in the alveolar septum (Fig. 2b, Masson’s trichrome). The lungs of BLM–treated mice (BLM + EtOH) displayed severe collagen deposition and fibrotic lesions. Accordingly, the Ashcroft score—used to assess the severity of lung fibrosis—was elevated following treatment with BLM compared to the control PBS+EtOH group (Fig. 2c). Notably, HSM pretreatment reduced both collagen deposition and the Ashcroft score in BLM–treated animals (BLM + HSM) (Fig. 2b, c).

We also performed BALF cell counts to evaluate the effects of HSM on the inflammatory response induced by BLM. Administration of BLM caused extensive infiltration of inflammatory cells, as shown by significant increases in total cells, macrophages, neutrophils, and lymphocytes (Fig. 2d–g). On the other hand, HSM reversed total cell and leukocyte accumulation in BALF of BLM–treated mice (Fig. 2d–g). Based on these results, we conclude that the HSM ethanol extract significantly decreases fibrosis and inflammatory cell infiltration in the lungs of BLM–treated mice.

### HSM reduces expression of TGF-β1, collagen 3α1, and α-SMA in lung tissues of BLM–treated mice

**Figure 3:**
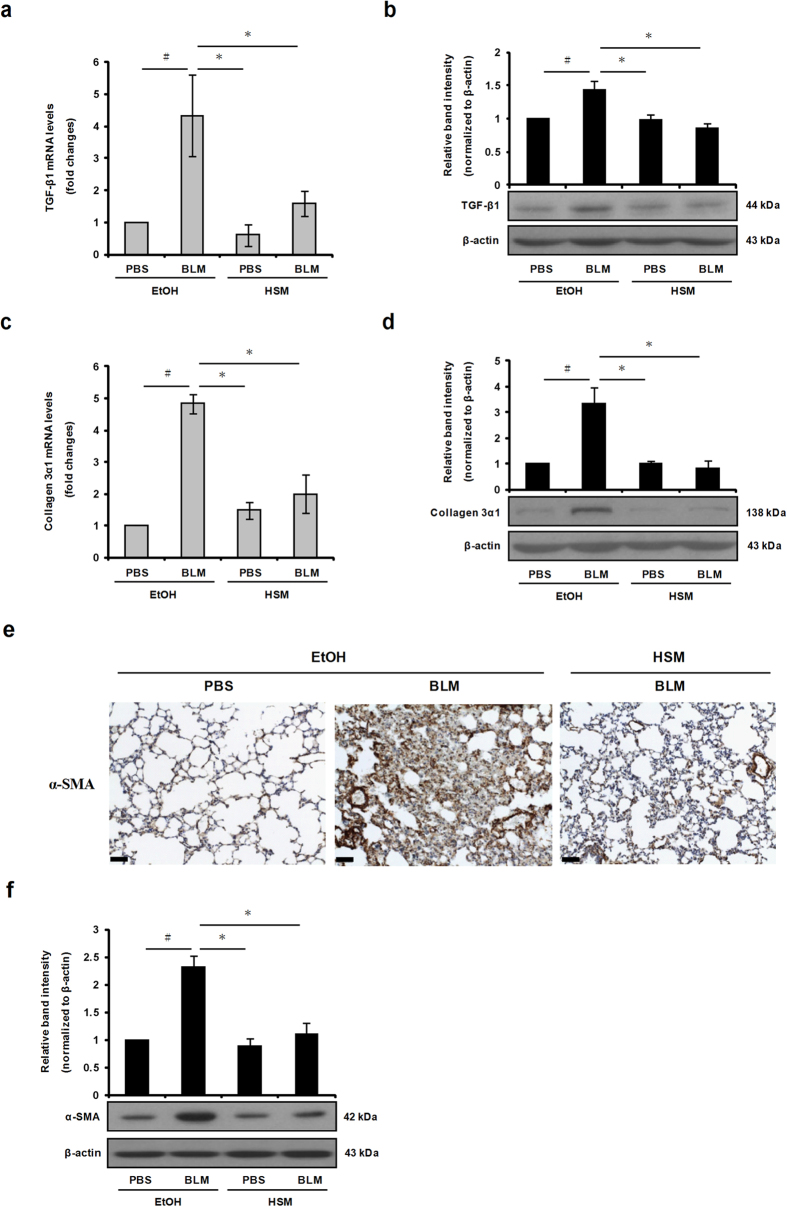
HSM inhibits TGF-β1, collagen 3α1, and α-SMA expression in lung tissues of BLM–treated mice. (**a,c**) mRNA levels of collagen 3α1 and TGF-β1 in lung tissues from each group of mice on day 21 were determined by quantitative real-time RT-PCR. All data are presented as fold changes of gene expression normalized to β-actin. (**b,d**) Western blotting detection of collagen 3α1 and TGF-β1 expression from lung tissues of each group of mice on day 21. Increased collagen 3α1 and TGF-β1 levels were observed in BLM–treated mice (BLM + EtOH) compared with control mice (PBS + EtOH). (**e**) Representative α-SMA staining of lung tissue sections from control and experimental groups at day 21. α-SMA-positive cells (myofibroblasts and airway smooth muscle cells) decreased in HSM-pretreated mice compared with BLM-treated mice (scale bars = 50 μm). (**f**) Western blotting detection of α-SMA expression from lung tissues of each group of mice on day 21. The blots were analyzed by densitometry and normalized to β-actin. Data are presented as means ± SEM of at least three separate experiments. ^#^*P* < 0.05 versus PBS + EtOH group. *P < 0.05 versus BLM + EtOH group.

The profibrotic cytokine TGF-β1 mediates the development of pulmonary fibrosis induced by BLM^10^. To evaluate the effects of HSM on TGF-β1 expression, we monitored TGF-β1 mRNA and protein expression levels in murine lung tissues using quantitative real-time RT-PCR and Western blotting. Our results showed that TGF-β1 mRNA and protein expression was induced by BLM (Fig. 3a,b). However, pretreatment with HSM significantly reduced TGF-β1 induction (Fig. 3a,b).

A previous study showed that collagen deposition in the BLM model of lung fibrosis is associated with enhanced expression of type III collagen^44^. We examined the effects of HSM on collagen 3α1 mRNA and protein expression in lung tissues of BLM–treated mice. Collagen 3α1 mRNA expression increased in lung tissues on day 7 following BLM treatment, whereas HSM treatment only slightly reduced collagen 3α1 expression (Supplementary Fig. S1a; day-7 histological lung sections are shown in Supplementary Fig. S2). On the other hand, HSM considerably reduced collagen 3α1 mRNA and protein expression in lung tissues of BLM-treated mice when examined on day 21 (Fig. 3c,d).

Next, we used α-SMA immunohistochemical staining to measure the extent of myofibroblast activation and fibrosis in lung sections. Lungs from BLM–treated mice displayed upregulated levels of α-SMA and increased numbers of α-SMA-positive cells, and these effects were significantly suppressed by HSM pretreatment (Fig. 3e). The protein expression of α-SMA was also detected via Western blotting in murine lung tissues. The expression level of α-SMA increased following BLM administration, while HSM pretreatment significantly reduced α-SMA expression on day 21 (Fig. 3f). These results indicate that HSM inhibits myofibroblast activation and collagen deposition in BLM–induced lung fibrosis.

### HSM attenuates the expression and production of IL-1β and IL-18 in lungs of BLM–treated mice

**Figure 4:**
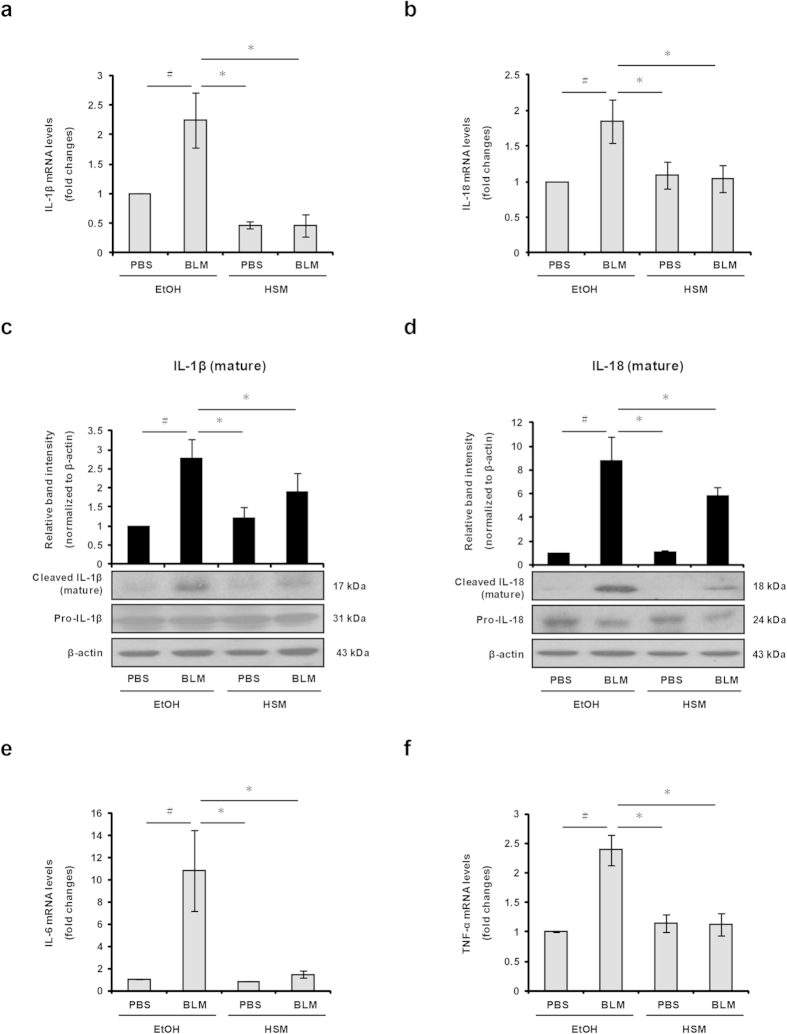
HSM reduces transcription and cleavage of IL-1β and IL-18 in lung tissues of BLM-treated mice. IL-1β (**a**) and IL-18 (**b**) mRNA expression levels in lung tissues from each group of mice on day 21 were examined by real-time RT-PCR. (**c,d**) Western blotting was performed to detect the cleaved products of IL-1β and IL-18 in lung tissues on day 21. Histograms show densitometry analysis of cleaved IL-1β and IL-18 normalized to β-actin. (**e,f**) Levels of IL-6 and TNF-α mRNA expression in lung tissues were examined on day 21 by real-time RT-PCR. Data are presented as means ± SEM. ^#^*P* < 0.05 versus PBS + EtOH group. *P < 0.05 versus BLM + EtOH group.

Both IL-1β and IL-18 play important roles in the pathogenesis of BLM–induced lung injury in animal models and humans^6^. To investigate whether HSM modulates the production of these proinflammatory cytokines, we examined their expression in the lungs of mice on day 7 and 21 using real-time RT-PCR. The mRNA level of IL-1β increased in lungs of BLM–treated mice sacrificed on day 7, and pretreatment with HSM appeared to prevent this effect; however, the effect of HSM did not reach statistical significance in this case (Supplementary Fig. S1b). In addition, HSM did not significantly affect the mRNA level of IL-18 compared with the control group (Supplementary Fig. S1c). On day 21, HSM prevented the induction effects of BLM on IL-1β and IL-18 expression (Fig. 4a,b). IL-1β and IL-18 protein expression in lungs was also assessed using Western blotting. Similar to mRNA results, the protein levels of cleaved IL-1β and IL-18 increased in BLM–treated mice (day 21), but HSM–pretreated mice showed significantly lower levels of the cleaved cytokines (Fig. 4c,d).

Previous studies have shown that the proinflammatory cytokines IL-6 and TNF-α also increase following BLM treatment^45^. As shown in Supplementary Fig. S1d and e, no statistically significant difference was noted in the mRNA levels of IL-6 and TNF-α on day 7. However, on day 21, the mRNA levels of both cytokines were reduced by HSM when compared to the BLM group (Fig. 4e,f). These results indicate that HSM regulates lung inflammation by inhibiting the expression of IL-1β, IL-18, IL-6, and TNF-α in BLM–treated mice.

### HSM inhibits BLM–induced caspase-1 activation and NLRP3 inflammasome expression in murine lungs

**Figure 5:**
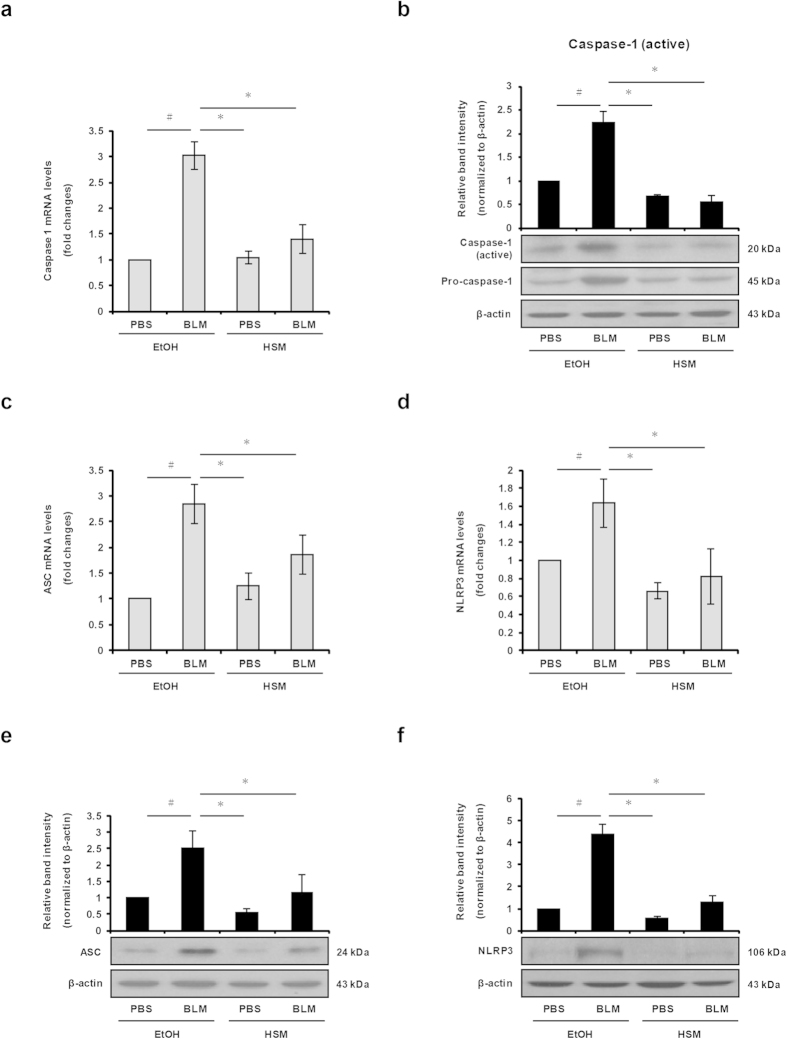
HSM suppresses BLM–induced activation of caspase-1 and expression of ASC and NLRP3 in lung tissues. (**a**) mRNA expression levels of caspase-1 in lung tissues were examined on day 21 by real-time RT-PCR. (**b**) Lung tissues from each group of mice were processed on day 21 for Western blotting to detect pro-caspase-1 p45 and caspase-1 subunit p20. (**c,d**) ASC and NLRP3 mRNA expression in mouse lung tissues were determined on day 21 by real-time RT-PCR. (**e,f**) Protein levels of ASC and NLRP3 in lung tissues were determined on day 21 by Western blotting. β-actin was used as a loading control. Data from three separate experiments are presented as means ± SEM. ^#^*P* < 0.05 versus PBS + EtOH group. *P < 0.05 versus BLM + EtOH group.

Caspase-1 expression and activation is required for the cleavage and secretion of IL-1β and IL-18 to their active forms^18^. To examine the effects of HSM on caspase-1 expression and activation, we measured mRNA and protein expression levels of caspase-1 in the lungs of mice. While no statistical effect was noticed for the samples taken on day 7 (Supplementary Fig. S1f), HSM-pretreated mice showed markedly lower levels of caspase-1 mRNA than BLM–treated mice when analyzed on day 21 (Fig. 5a). Levels of active caspase-1 protein were significantly increased in the lungs of BLM–treated mice on day 21; however, HSM significantly reduced active caspase-1 protein levels under these conditions (Fig. 5b).

The NLRP3 inflammasome is formed by three components that include the NOD-like receptor NLRP3, the adaptor molecule ASC, and pro-caspase-1^18^. We examined whether HSM decreases BLM–induced IL-1β and IL-18 production by regulating the expression of ASC and NLRP3. As shown in Supplementary Fig. S1g and h, ASC and NLRP3 mRNA levels were elevated in BLM-treated mice compared to the control PBS+EtOH group (day 7). While HSM showed a tendency to reduce ASC and NLRP3 mRNA levels compared to the BLM+EtOH group on day 7, the effect was not statistically significant (Supplementary Fig. S1g, h). In contrast, HSM reduced ASC and NLRP3 mRNAs in a statistically significant manner in the lungs of BLM–treated mice when examined on day 21 (Fig. 5c,d). Similar results were obtained for the ASC and NLRP3 proteins when assessed on day 21 (Fig. 5e,f).

**Figure 6:**
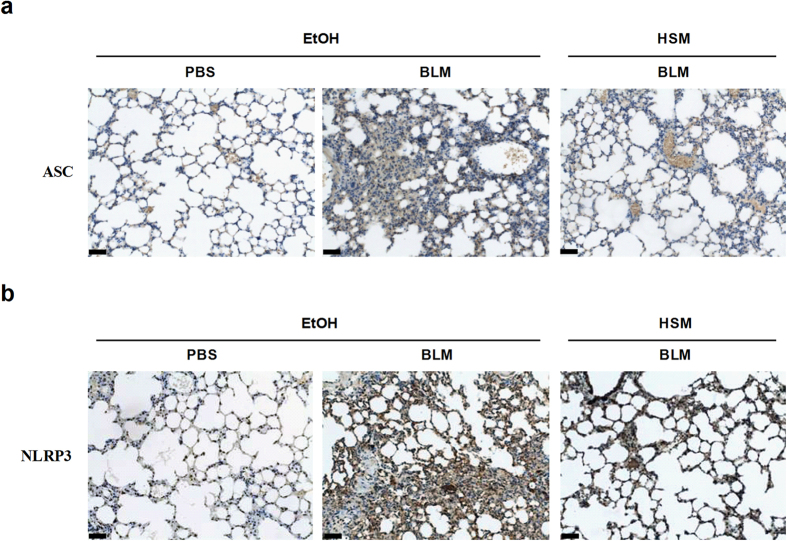
Immunohistochemical staining of ASC and NLRP3 in lungs of BLM-treated mice. Lung tissues from mice were obtained 21 days after BLM administration and cross-sections of left lung tissues were stained for ASC (**a**) or NLRP3 (**b**). Representative images show expression of ASC and NLRP3 in alveolar macrophages and lung epithelial cells. Increased ASC and NLRP3 expression was observed in lungs of BLM–treated mice (BLM + EtOH), and expression of ASC and NLRP3 was significantly reduced in lungs of HSM–pretreated mice (BLM + HSM). Scale bars=50 μm.

Immunohistochemistry analysis also demonstrated that HSM pretreatment could attenuate BLM–induced expression of ASC and NLRP3 in lung tissues (Fig. 6a,b). Based on these results, we suggest that the HSM ethanol extract may attenuate BLM–induced lung inflammation by regulating the NLRP3 inflammasome and the production of IL-1β and IL-18.

### HSM inhibits BLM–induced P2X_7_R expression and ROS production

**Figure 7:**
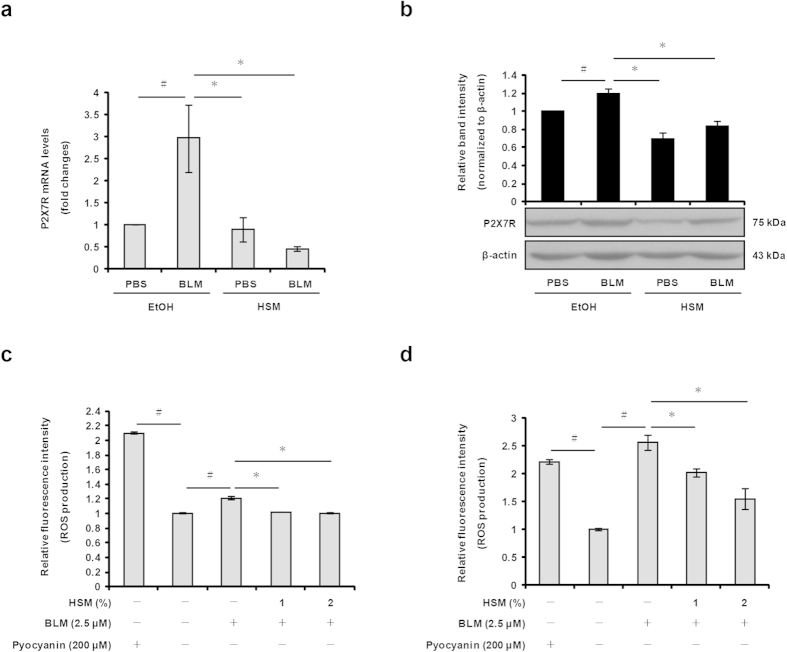
Inhibitory effects of HSM on BLM–induced P2X_7_R expression and ROS production. (**a**) P2X_7_R mRNA expression levels in mouse lung tissues were measured on day 21 by real-time RT-PCR. (**b**) P2X_7_R protein expression in lung tissues was analyzed on day 21 by Western blotting. ROS production in MLE12 cells (**c**) and MRC-5 cells (**d**) was measured using a ROS detection kit and a fluorescence microplate reader. Pyocyanin (200 μM) was used as a positive control for ROS formation. Data are representative of three separate experiments and are expressed as means ± SEM. ^#^*P* < 0.05 versus PBS+EtOH group or untreated (ethanol-treated) cells. *P < 0.05 versus BLM + EtOH group or control (BLM–treated) cells.

Recent studies have shown that BLM administration results in a large increase of ATP release and P2X_7_R protein expression in lung epithelial cells^28^,^46^. ATP binding to the purinergic receptor P2X_7_R activates the NLRP3 inflammasome^18^. To examine whether HSM affects the expression of P2X_7_R in the lungs, we performed real-time RT-PCR and Western blotting analyses to examine P2X_7_R mRNA and protein expression. The levels of P2X_7_R mRNA and protein significantly increased in the lungs of BLM–treated mice (Fig. 7a,b). Notably, the increase of P2X_7_R mRNA and protein expression was largely prevented by HSM pretreatment (Fig. 7a,b).

Activation of P2X_7_R by ATP increases the production of ROS, which are involved in some cases in inflammasome activation and secretion of IL-1β and IL-18^20^,^47^. ROS are not only produced in response to BLM but are also thought to contribute to the development of pulmonary fibrosis^25^. To ensure that the inhibition of ROS production was not due to cytotoxic effects produced by HSM, we cultured murine MLE12 lung epithelial cells for 48 h with different concentrations of HSM extract (1, 2 and 5%), prior to monitoring cell viability using the MTT assay. While a significant decrease of viability was observed in cells treated with 5% HSM compared with control ethanol–treated cells, the HSM extract produced no cytotoxic effect at 1% or 2% (data not shown). We used a fluorescence detection kit to examine the production of intracellular ROS in MLE12 lung epithelial cells and MRC-5 lung fibroblasts pretreated with HSM extract (1 or 2%) for 24 h, prior to treatment with BLM (2.5 μM) for 24 h. As shown in Fig. 7c,d, treatment of MLE12 lung epithelial cells and MRC-5 lung fibroblasts with BLM increased ROS production; however, pretreatment with HSM significantly reduced ROS production under these conditions. Taken together, these results show that HSM inhibits NLRP3 inflammasome activation in the lungs of BLM–treated mice, at least in part, by reducing P2X_7_R expression and ROS production.

### HSM enhances SOD expression

**Figure 8:**
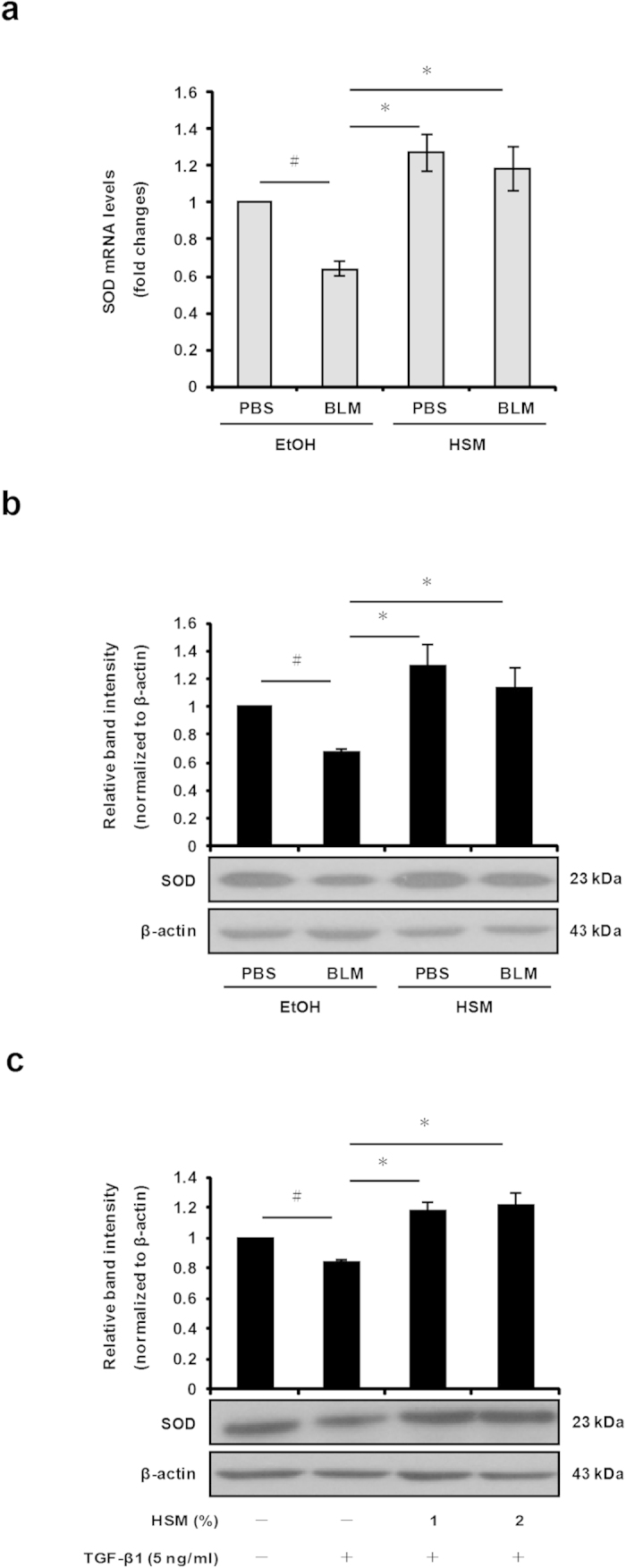
HSM restores SOD expression in the lungs of BLM-treated mice and lung fibroblasts. (**a**) SOD mRNA expression in the lung tissues was measured on day 21 by real-time RT-PCR. Data are presented as fold changes in gene expression normalized to β-actin. (**b**) SOD protein expression in the lung tissues was measured on day 21 by Western blotting. Quantitative results are from three independent experiments. (**c**) Western blotting of SOD protein in MRC-5 cells pretreated with 1% or 2% of HSM extract, prior to treatment with TGF-β1 (5 ng/ml) for 24 h. Relative protein levels were quantified by densitometry and normalized to β-actin. ^#^*P* < 0.05 versus PBS + EtOH group or untreated (ethanol-treated) cells. *P < 0.05 versus BLM + EtOH group or control cells (TGF-β1–treated).

SOD catalyzes the breakdown of superoxide radicals into oxygen and hydrogen peroxide in the cytoplasm, thereby acting as a potent antioxidant enzyme^26^. Decreased SOD expression has been observed in BLM-treated animals with pulmonary fibrosis^27^. To evaluate whether HSM pretreatment can modulate SOD expression in the lungs of BLM–treated mice, we examined SOD mRNA and protein expression in lung tissues. On day 21, SOD mRNA and protein levels were significantly reduced in the lungs of BLM-treated mice (BLM+EtOH) compared with control mice (PBS+EtOH) (Fig. 8a,b). However, pretreatment with HSM increased SOD mRNA and protein expression to levels similar to that of the control PBS group (Fig. 8a,b).

TGF-β1 has been shown to suppress the expression of SOD in a previous study^48^. We examined the effects of HSM on TGF-β1–regulated SOD expression in human MRC-5 lung fibroblasts. SOD protein expression was markedly reduced in human lung fibroblasts treated with recombinant human TGF-β1 (Fig. 8c). Notably, pretreatment with HSM restored SOD protein levels in TGF-β1–treated cells (Fig. 8c). These findings suggest that the inhibitory effects of HSM on ROS production may be due at least in part to modulation of SOD expression.

## Discussion

The most studied fibrosis model is based on the administration of BLM to laboratory animals, a process which induces lung injury through two distinct phases. The first phase involves inflammation of the lungs and is characterized by an influx of inflammatory cells, in particular, macrophages, neutrophils, and lymphocytes. The second phase involves lung fibrosis which is characterized by ECM remodeling and extensive collagen deposition^49^.

We show here that an ethanol extract of HSM produces anti-fibrotic activities as shown by the inhibition of TGF-β1-induced myofibroblast differentiation and myofibroblast marker expression (α-SMA and fibronectin) in human MRC-5 lung fibroblast cells. TGF-β signaling is essential for profibrotic processes which include fibroblast activation, differentiation into myofibroblasts, and ECM deposition. The signaling pathways activated by TGF-β consist of Smad-dependent and Smad-independent signaling pathways^12^,^13^. The Smad-dependent signaling pathway is necessary but not sufficient for a full response to TGF-β. Previous studies have shown that TGF-β1-induced α-SMA and fibronectin expression may be partially mediated by the PI3K/Akt pathway^12^,^13^. In the present study, we first determined that the inhibitory effects of HSM on TGF-β1–mediated α-SMA and fibronectin expression occurs through reduction of Smad-dependent (Smad2/3) and Smad-independent (Akt) signaling transduction in human lung fibroblasts. We also verified the effects of HSM in the murine model of BLM–induced pulmonary fibrosis *in vivo*. Administration of BLM caused destruction of the lung architecture and led to pulmonary fibrosis characterized by increased TGF-β1 expression and collagen deposition in the lungs. Pretreatment with HSM reduced the biochemical and histological signs of lung fibrosis, including collagen deposition, and α-SMA, TGF-β1 expression. These data are in agreement with previous studies which demonstrated that down-regulation of α-SMA, collagen and TGF-β1 expression, and inhibition of TGF-β1/Smad signaling pathways are involved in mediating the effects of *C. sinensis* against renal and liver fibrosis^36^,^37^,^38^. On the other hand, we cannot rule out the possibility that the anti-fibrotic effects of HSM may also involve decreased expression of the TGF receptor in lung fibroblasts.

The pathogenesis of IPF begins with alveolitis which is characterized by the accumulation of inflammatory cells within lung parenchyma. Neutrophils and mononuclear cells accumulate and cytokines (e.g., TGF-β1) are released to induce fibroblast proliferation and migration into the areas of acute lung injury, eventually leading to enhanced secretion of collagen and other ECM proteins^50^. We found that HSM produces anti-inflammatory activities *in vivo*, as shown by reduced inflammatory cell counts in BALF following BLM administration, and reduced IL-1β and IL-18 expression levels in the lungs of BLM-treated mice. Our findings suggest that HSM inhibits the infiltration of inflammatory cells into lung tissues, which is an important anti-inflammatory effect in this murine model of BLM-induced lung fibrosis. Consistent with these results, our previous study showed that HSM inhibits ATP–induced IL-1β and IL-18 secretion in LPS–primed macrophages^35^.

Several cytokines such as IL-6 and TNF-α are thought to promote the development of lung fibrosis^8^,^51^. We found that lung IL-6 and TNF-α levels were significantly increased after BLM administration in the BLM–induced murine pulmonary fibrosis model. HSM significantly suppressed the production of IL-6 and TNF-α in the lungs, possibly contributing to its anti-inflammatory effects. In addition, IL-1β–dependent IL-6 upregulation plays a critical role in fibroblast activation and proliferation in human acute lung injury^52^. IL-6 trans-signaling activates canonical TGF-β signaling via Smad 3 activation which leads to collagen expression in dermal fibroblasts^53^. Moreover, both IL-1β and TNF-α treatment induced the secretion of large amounts of TGF-β1 in rat pulmonary artery endothelial cells^54^. Therefore, our observation that HSM inhibits BLM–induced pulmonary injury and TGF-β1 expression may occur through down-regulation of IL-1β, IL-6, IL-18, and TNF-α.

It has been reported that BLM-induced IL-1β production and lung inflammation are dependent on the inflammasome-adaptor protein, ASC^55^. Furthermore, uric acid released from injured cells exposed to BLM represents a major danger signal that activates the NLRP3 inflammasome, leading to IL-1β production and lung inflammation and fibrosis^56^. These authors also found that BLM-induced enhancement of lung collagen production was attenuated in mice lacking either NLRP3 or caspase-1, and that NLRP3– and ASC–deficient mice were resistant to BLM-induced skin and lung fibrosis. Inhibition of caspase-1 in dermal and lung fibroblasts significantly inhibited the expression of IL-1β, IL-18, collagen, and α-SMA^23^. Recently, TGF-β signaling and Smad2/3 activation in renal tubular epithelial cells was reported to depend on NLRP3 independently of its ability to form a caspase-1–activating inflammasome^24^. NLRP3 expression increased in response to TGF-β1 stimulation and was associated with expression of the myofibroblast marker α-SMA. Furthermore, TGF-β1–induced expression of α-SMA significantly decreased in NLRP3–deficient renal tubular epithelial cells. Taken together, these findings suggest the involvement of the NLRP3 inflammasome in BLM–induced lung inflammation and fibrosis. We observed that pretreatment with HSM extract significantly reduced caspase-1 activation and NLRP3 and ASC expression in the lungs of BLM–treated mice. This observation correlated with decreased levels of IL-1β, IL-18 and TGF-β1 in the lungs of HSM–pretreated mice.

Prior studies have demonstrated that BLM–induced pulmonary fibrosis is associated with marked increase of ROS production^11^,^25^. In pulmonary fibrosis, TGF-β activation is considered a hallmark of disease progression^57^. *In vitro* studies have shown that ROS enhance the release of TGF-β1 from human alveolar epithelial cells^58^. TGF-β1 also increases ROS production through activation of NADPH oxidase in human lung fibroblasts^59^. Recently it was shown that mitochondria-derived ROS can activate the NLRP3 inflammasome^18^, and that ATP–induced P2X_7_R activation promotes the rapid production of ROS, which in turn activate the NLRP3 inflammasome^47^. Thus, inhibition of ROS production and P2X_7_R expression might play a role in the anti-fibrotic and anti-inflammatory activities of HSM extract in BLM–induced pulmonary fibrosis. We observed that BLM caused a significant increase in the production of ROS in lung epithelial cells. Of note, pretreatment of the cells with HSM can inhibit BLM–induced ROS production. We found that HSM pretreatment can also inhibit P2X_7_R expression in the lungs of BLM–treated mice. Similar observations were made in our previous study as HSM inhibited P2X_7_R expression and ROS production in LPS–primed macrophages^35^, indicating that the HSM ethanol extract may have the general ability to reduce inflammation in different mammalian cells.

In mammals, there are three different SODs: intracellular copper-zinc SOD (Cu/Zn-SOD), mitochondrial manganese SOD (Mn-SOD), and extracellular SOD (EC-SOD); these enzymes can be detected in all classes of lung cells^26^. Administration of lecithinized SOD (PC-SOD), a synthesized lecithinized human recombinant Cu/Zn-SOD, can significantly attenuate BLM–induced pulmonary inflammatory responses and fibrosis in mice^14^. In addition, mice lacking EC-SOD display a marked increase in lung inflammation and fibrosis in response to BLM^60^. In the present study, BLM induced a significant decrease in SOD expression, and pretreatment with HSM prevented the inhibitory effects of BLM and increased SOD levels. These findings suggest that the decreased levels of SOD in the lungs of BLM–treated mice may produce oxidative stress that further promotes the fibrotic response, whereas the HSM ethanol extract can inhibit oxidation and alleviate lung fibrosis by increasing SOD expression.

**Figure 9:**
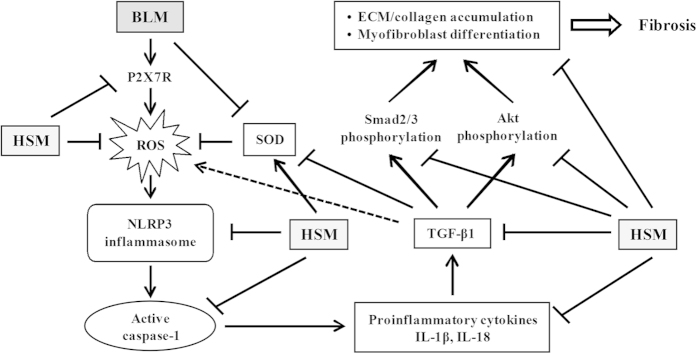
Diagram illustrating the mechanisms of action of HSM ethanol extract in inhibiting the development of BLM-induced pulmonary inflammation and fibrosis. HSM inhibits the production of proinflammatory and profibrotic cytokines, P2X_7_R expression, ROS production, NLRP3 inflammasome activation, and TGF-β1-mediated Smad-dependent and Smad-independent signaling pathways, in addition to stimulating the production of SOD in the experimental model of lung fibrosis.

In conclusion, we demonstrate that the HSM ethanol extract can efficiently ameliorate BLM–induced lung inflammation and fibrosis by inhibiting infiltration of inflammatory cells and the production of immune mediators in mice. This protective effect is based on reduced expression of TGF-β1, collagen 3α1, fibronectin, and α-SMA; decreased pro-inflammatory cytokines, such as IL-1β, IL-18, IL-6, and TNF-α; inhibition of NLRP3 inflammasome activation; decreased expression of P2X_7_R; inhibition of ROS production; elevation of antioxidant enzyme such as SOD; and inhibition of Smad2/3 and Akt phosphorylation (Fig. 9). These results suggest that the HSM ethanol extract may be used to prevent and treat pulmonary fibrosis. In view of the existing similarities between the mechanisms responsible for the development of fibrosis in the lungs and in other organs such as the liver and kidneys^36^,^37^,^38^, it is tempting to speculate that the HSM extract may also produce beneficial effects for these conditions as well.

## Additional Information

**How to cite this article**: Huang, T.-T. *et al.* *Hirsutella sinensis* mycelium attenuates bleomycin-induced pulmonary inflammation and fibrosis *in vivo*. *Sci. Rep.* **5**, 15282; doi: 10.1038/srep15282 (2015).

## Supplementary Material

Supplementary Information

## Figures and Tables

**Table 1:** Primer sequences used for quantitative real-time RT-PCR.

Gene	Forward primer (5′–3′)	Reverse primer (5′–3′)
ASC	GAGCTGCTGACAGTGCAAC	GCCACAGCTCCAGACTCTTC
Caspase-1	ACCCTCAAGTTTTGCCCTTTAGAA	TCTGAGGTCAACTTGGACTCCAAC
Collagen 3α1	GTTCTAGAGGATGGCTGTACTAAACACA	TTGCCTTGCGTGTTTGATATTC
IL-1β	GCTCATCTGGGATCCTCTCC	CCTGCCTGAAGCTCTTGTTG
IL-6	CCACTTCACAAGTCGGAGGCTTA	GCAAGTGCATCATCGTTGTTCATAC
IL-18	ACAACTTTGGCCGACTTCAC	GGGTTCACTGGCACTTTGAT
NLRP3	AGAGCCTACAGTTGGGTGAAATG	CCACGCCTACCAGGAAATCTC
P2X_7_R	TCTTCCGACTAGGGGACATCT	ATGGGACCAGCTGTCTAGGTT
SOD	GCAGGGAACCATCCACTT	TGCCCAGGTCTCCAACAT
TGF-β1	AAACGGAAGCGCATCGAA	GGGACTGGCGAGCCTTTAGTT
TNF-α	GTGGAACTGGCAGAAGAGGC	AGACAGAAGAGCGTGGTGGC
β-actin	GATTACTGCTCTGGCTCCTAGC	GACTCATCGTACTCCTGCTTGC
