# Supplementary material for: Hirsutella sinensis mycelium attenuates bleomycin-induced pulmonary inflammation and fibrosis in vivo
Source: Sci Rep. 2015 Oct 26;5:15282. doi: 10.1038/srep15282 (PMC4620496; doi:10.1038/srep15282)
Supplement: Supplementary Information [file srep15282-s1.pdf]

Supplementary Material

Hirsutella sinensis mycelium attenuates bleomycin-induced pulmonary inflammation and fibrosis *in vivo*

Tsung-Teng Huang[1,2,3], Hsin-Chih Lai[1,2], Yun-Fei Ko[4,5], David M. Ojcius[1,6], Ying-Wei Lan[7], Jan Martel[1,3], John D. Young[1,3,4,5,8]*, Kowit-Yu Chong[2,7,9]*

[1]Center for Molecular and Clinical Immunology, College of Medicine, Chang Gung University, Taoyuan 33302, Taiwan, ROC, [2]Department of Medical Biotechnology and Laboratory Science, College of Medicine, Chang Gung University, Taoyuan 33302, Taiwan, ROC, [3]Laboratory of Nanomaterials, College of Medicine, Chang Gung University, Taoyuan 33302, Taiwan, ROC, [4]Biochemical Engineering Research Center, Ming Chi University of Technology, New Taipei City 24301, Taiwan, ROC, [5]Chang Gung Biotechnology Corporation, Taipei 10508, Taiwan, ROC, [6]Department of Biomedical Sciences, University of the Pacific, Arthur Dugoni School of Dentistry, San Francisco, CA 94103, USA, [7]Graduate Institute of Biomedical Sciences, Division of Biotechnology, College of Medicine, Chang Gung University, Taoyuan 33302, Taiwan, ROC, [8]Laboratory of Cellular Physiology and Immunology, Rockefeller University, New York, NY 10021, USA, [9]Department of Family Medicine, Chang Gung Memorial Hospital-Linkou, Taoyuan 33302, Taiwan, ROC.

*Correspondence and requests for materials should be addressed to J.D.Y. (dingeyoung@hotmail.com) or K.-Y.C. (kchong@mail.cgu.edu.tw)

Supplementary Figures

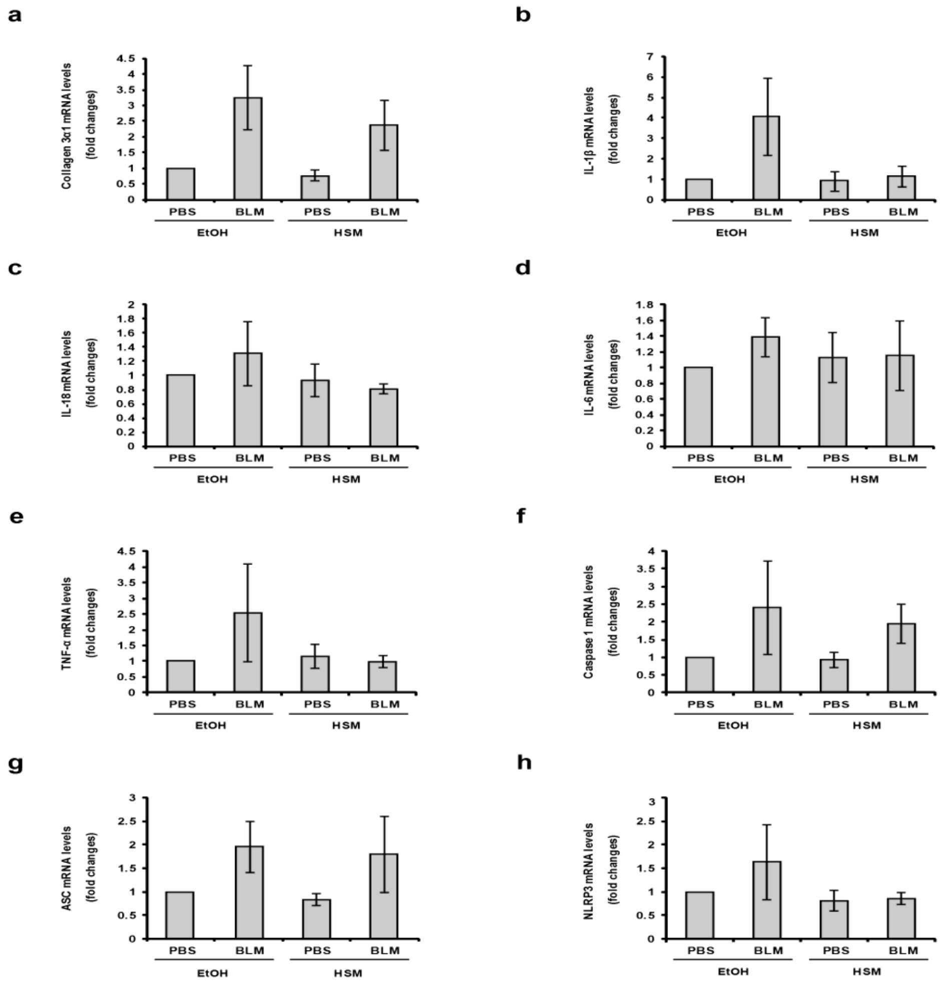

Figure S1｜Effects of HSM pretreatment on BLM-induced inflammation and fibrosis-related gene expression in lung tissues on day 7. Lung tissues were subjected to quantitative real-time RT-PCR to detect the effects of HSM on mRNA expression of collagen 3α1 (a), IL-1β (b), IL-18 (c), IL-6 (d), TNF-α (e), caspase-1 (f), ASC (g), and NLRP3 (h). Data are presented as fold change in gene expression normalized to β-actin. Results are expressed as means ± SEM. The number of mice analyzed in each group ranged from 3 to 5.

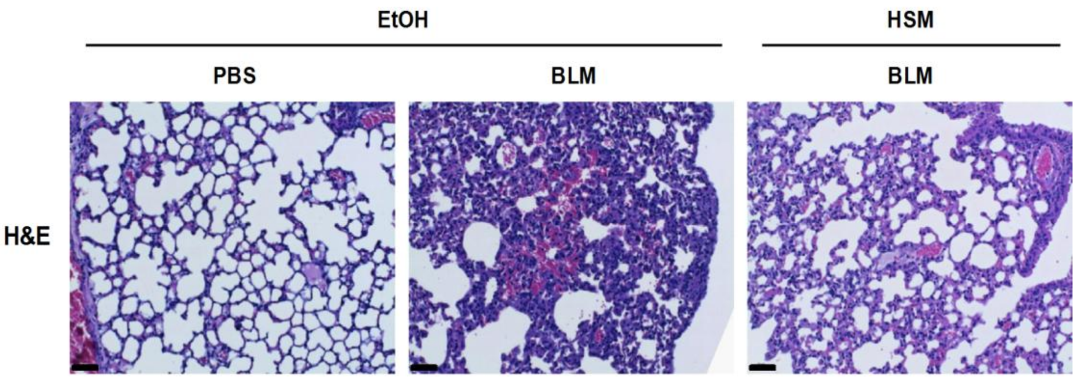

Figure S2｜Representative histological lung sections of BLM/HSM-treated mice. Paraffin sections from lung tissues of the mice on day 7 were stained with H&E. Scale bars＝50 μm.
